# Viral Reprogramming of Nucleotide Synthesis and Its Impact on Viral Infection

**DOI:** 10.1002/jmv.70563

**Published:** 2025-08-20

**Authors:** Lara Dsouza, Zhilong Yang

**Affiliations:** ^1^ Department of Veterinary Pathobiology, College of Veterinary Medicine & Biomedical Sciences Texas A&M University College Station Texas USA

**Keywords:** antiviral immune responses, nucleotide metabolism, purine, pyrimidine, signaling, viral infection, virus

## Abstract

Nucleotides are the building blocks of DNA and RNA. They also play essential roles in various other biological processes, including protein glycosylation, ribosome biogenesis, and cytoskeletal function. The significance of the regulation of nucleotide metabolism has recently gained more attention in many physiological and pathological contexts, including viral infections that often reprogram host cell metabolism to support viral replication. However, whilst nucleotides have long been known to be important for viral nucleic acid synthesis, the molecular mechanisms undertaken by viruses to regulate nucleotide synthesis are only beginning to be understood. In this review, we present a comprehensive analysis of nucleotide regulation by upstream growth factor signaling mechanisms in various families of RNA and DNA viruses, such as herpesviruses, poxviruses, influenza viruses, and coronaviruses. We place a primary emphasis on discussing the signaling pathways as the regulatory mechanisms and highlight the gaps in understanding the mechanistic details. We underscore recent research that investigates the roles of different viral factors in modulating nucleotide metabolism in the infections of DNA and RNA viruses. Finally, we discuss the emerging area of inquiry that explores the relationship between nucleotide metabolism and immune regulation. A thorough understanding of how nucleotides are regulated during viral infections is essential for developing novel effective therapeutic strategies against these viruses.

## 1 Introduction

Nucleotides are integral to living cells, serving as the essential components of DNA and RNA. Each nucleotide is structurally composed of a pentose sugar (either ribose or deoxyribose), a nitrogenous base (which can be categorized as either a purine or a pyrimidine), and a phosphate group. Adenine and guanine are the two primary purine bases found in both DNA and RNA. In contrast, cytosine is the only pyrimidine base identified within both types of nucleic acids. DNA contains thymine as its other pyrimidine, whereas RNA is characterized by the presence of uracil [1, 2, 3]. Nucleotides are fundamental to cellular processes and serve as essential cofactors in various metabolic pathways. They facilitate the release of free energy through the hydrolysis of high‐energy ATP compounds and play critical roles in multiple key biological functions, including protein glycosylation, ribosome biogenesis, as well as DNA replication and repair [4].

Cells utilize two distinct mechanisms to provide nucleotides for their metabolic demands: the de novo pathways and the salvage pathways. The de novo pathways pertain to the synthesis of purine and pyrimidine rings from fundamental molecular components, including amino acids, ribose‐5‐phosphate, carbon dioxide, and ammonia (NH_3_). Conversely, the salvage pathways recycle nucleobases and nucleic acids that result from the degradation of pre‐existing nucleic acids [5]. The specific mechanism employed depends on the cell's prevailing energy and metabolic demands.

Rapidly proliferating cells, along with those exhibiting increased metabolic demands, such as virus‐infected cells, rely on the de novo pyrimidine biosynthesis pathway to fulfill their increased nucleotide requirements.

**Figure 1:**
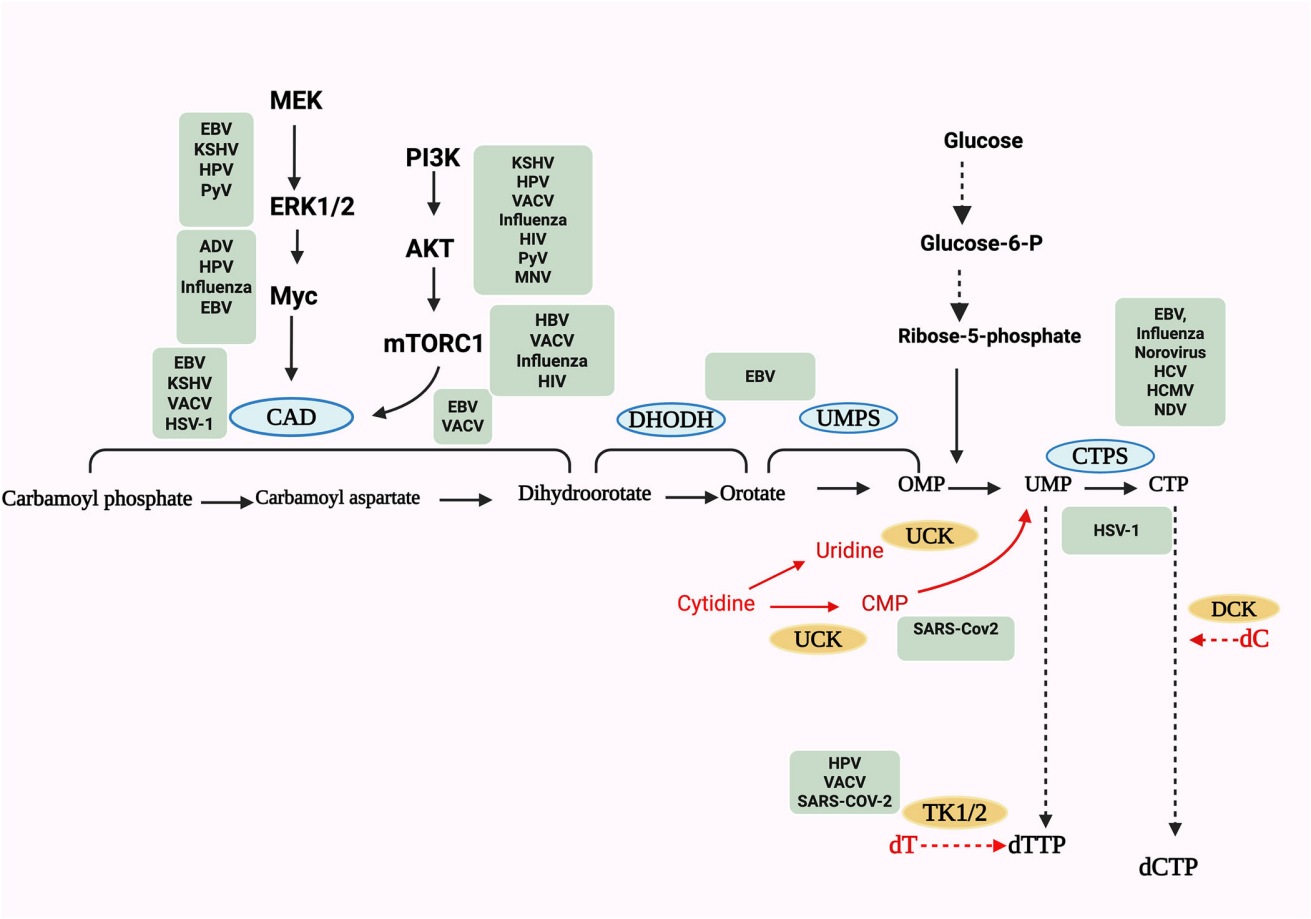
The regulation of pyrimidine metabolism by various viruses. The de novo pyrimidine synthesis is highlighted in black and salvage pathways for pyrimidine metabolism are highlighted in red. Signaling and feeder pathways are highlighted in bold. Viruses regulating different nodes of pyrimidine metabolism are named in green boxes near the respective signaling cascade or enzymes. We have attempted to cover most viruses known to not only regulate nucleotide metabolism directly but also influence signaling and feeder pathways. For certain viruses, studies have shown that they activate regulatory pathways; however, an established link to nucleotide metabolism, while promising, has yet to be confirmed. ADV, adenovirus (adenovirus family); CAD, carbamoyl phosphate synthetase II, aspartate transcarbamoylase and dihydroorotase; CTP, cytidine triphosphate; dC, deoxycytidine; DHODH, dihydroorotate dehydrogenase; dT, deoxythymidine; dTTP, deoxythymidine triphosphate; EBV, Epstein–Barr virus (herpesvirus family); HCMV, human cytomegalovirus (cytomegalovirus family); HCV, hepatitis C virus (flavivirus family); HIV, Human Immunodeficiency virus (retrovirus family); HPV, human papillomavirus (papillomavirus family); KSHV, Kaposi's sarcoma‐associated herpesvirus (herpesvirus family); MNV, murine norovirus (calicivirus family); NDV, Newcastle disease virus (paramyxovirus family); PyV, polyoma virus (polyomavirus family); Sars‐COV‐2, Severe Acute Respiratory Syndrome Coronavirus 2(Coronavirus family); TK1/2, thymidine kinase ½; UCK, uridine‐cytidine kinase1/2; UMPS, UMP synthase; VACV, vaccinia virus (poxvirus family).

The de novo pyrimidine biosynthesis pathway commences with the formation of a pyrimidine ring, which subsequently pairs with ribose‐5‐phosphate, a component derived from the pentose phosphate pathway (PPP). Uridine monophosphate (UMP), the first precursor of pyrimidines, is produced through a series of six enzymatic reactions involving three principal enzymes of this pathway. The initial three steps are catalyzed by the enzyme CAD, which encompasses carbamoyl phosphate synthetase II, aspartate transcarbamoylase, and dihydroorotase. These reactions yield dihydroorotate, which is then converted to orotate through the action of DHODH. Subsequently, orotate undergoes further enzymatic conversion to form orotate‐5‐monophosphate, and ultimately, UMP through the enzyme uridine monophosphate synthetase (UMPS). Following phosphorylation, UMP can be converted to cytidine triphosphate (CTP) via the enzyme CTP synthase [6, 7, 8, 9] (Figure 1).

When the metabolic demands of the cell are low, such as in resting cells and differentiated nonmalignant cells, the pyrimidine supply is derived from the degradation of DNA and RNA. The salvage pathway facilitates the uptake of free pyrimidines, which are subsequently converted into nucleoside or deoxynucleoside monophosphates (NMPs/dNMPs). Thymidine kinases 1 and 2 are responsible for the conversion of deoxythymidine to deoxythymidine monophosphate, while deoxycytidine kinase catalyzes the conversion of deoxycytidine‐to‐deoxycytidine monophosphate (dCMP). The enzymes cytidine deaminase and uridine‐cytidine kinase (UCK) are responsible for the conversion of deoxycytidine to uracil and UMP [5, 9, 10, 11] (Figure 1).

**Figure 2:**
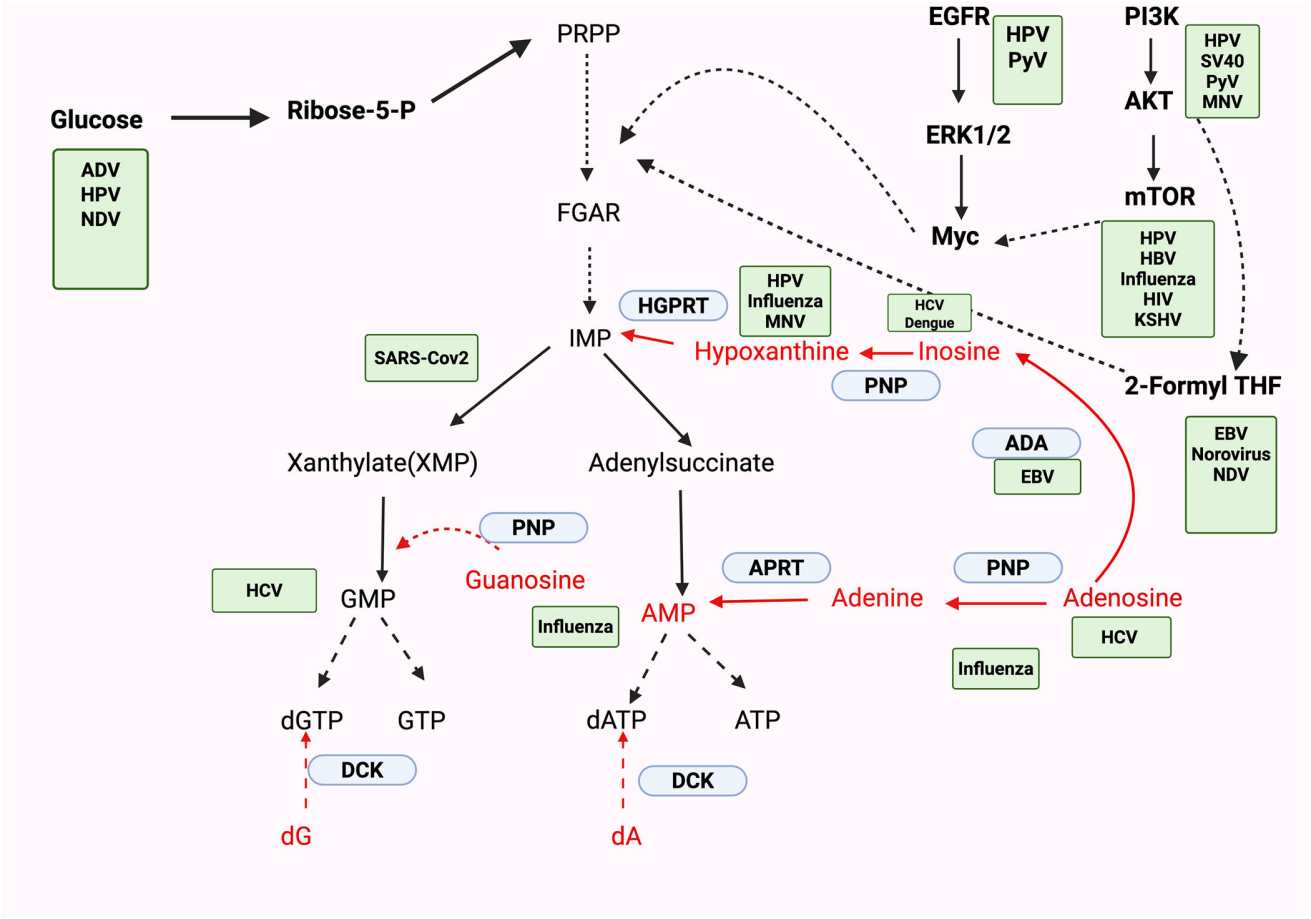
The regulation of purine metabolism by various viruses. The de novo purine synthesis is highlighted in black and salvage pathways for purine metabolism is highlighted in red. Signaling and feeder pathways are highlighted in bold. Viruses are regulating different nodes of purine metabolism are named in green boxes near the respective signaling cascade or enzyme. We have attempted to cover most viruses known to not only regulate nucleotide metabolism directly but also influence signaling and feeder pathways. For certain viruses, studies have shown that they activate regulatory pathways; however, an established link to nucleotide metabolism, while promising, has yet to be confirmed. ADA, adenosine deaminase; ADV, adenovirus (adenovirus family); APRT, adenine phosphoribosyl transferase; ATP, adenosine triphosphate; dA, deoxyadenosine; dATP, deoxyadenosine triphosphate; DCK, deoxycytidine kinase; dG, deoxyguanosine; dGTP, deoxyguanosine triphosphate; EBV, Epstein–Barr virus (herpesvirus family); FGAR, phosphoribosyl‐N‐formylglycineamide; GTP, guanosine triphosphate; HCV, hepatitis c virus (flavivirus family); HGPRT, hypoxanthine‐guanine phosphoribosyl transferase; HPV, human papillomavirus (papillomavirus family); IMP, inosine monophosphate; MNV, murine norovirus (calicivirus family); NDV, Newcastle disease virus (paramyxovirus family); PNPL, purine nucleoside phosphorylase; PRPP, phosphoribosyl pyrophosphate; PyV, polyoma virus (polyomavirus family); Sars‐COV‐2, Severe Acute Respiratory Syndrome Coronavirus 2 (Coronavirus family).

Purine synthesis is a complex process that encompasses a series of 10 reactions, requiring several key substrates, including glutamine, glycine, bicarbonate, and N‐10 formyl‐tetrahydrofolate (THF). The ultimate product of this pathway, inosine monophosphate (IMP), can be further converted into either adenosine monophosphate (AMP) or guanosine monophosphate (GMP) through distinct enzymatic reactions. The synthesis of AMP occurs via adenylo‐succinate, facilitated by the enzymes adenylosuccinate synthetase and adenylosuccinate lyase. Conversely, GMP is produced through a xanthylate intermediate, which involves the action of IMP dehydrogenase and XMP‐glutamine amidotransferase [5]. The salvage pathway involves the catabolism of adenosine, guanosine, and inosine to nucleobases adenine, guanine, and hypoxanthine, respectively, by purine nucleoside phosphorylase and then further converted to NMPs through adenine phosphoribosyl transferase or hypoxanthine‐guanine phosphoribosyl transferase. In addition, deoxyadenosine and deoxyguanosine can be converted to dAMP or dGMP by DCKs [9, 10, 12] (Figure 2).

Viruses, as intracellular parasites without metabolism, exploit the metabolic resources of their host cells to facilitate their own replication [40, 41, 42, 43]. Nucleotide analogs or inhibitors that target nucleotides have been a longstanding area of interest in the development of antiviral agents. To date, over 30 nucleoside analogs have received approval from the U.S. Food and Drug Administration, and their safety profiles and efficacy have been thoroughly studied [44]. Research into the molecular mechanisms governing nucleotide synthesis in the context of viral infection is beginning to gain traction; however, many of these molecular processes remain poorly understood.

The regulation of nucleotides is highly controlled and regulated in response to environmental cues and metabolic requirements of the cell. Nucleotides are regulated at the transcriptional level through transcription factors and signaling, allosteric regulation through enzymes, and indirect regulation through certain metabolites [2, 5]. Growth factor signaling and biosynthetic pathways are generally altered in virus‐infected cells, with increasing evidence of major regulators of these pathways that are usurped to increase nucleotide synthesis for efficient virus replication [41]. In this review, we primarily focus on the signaling pathways such as mTOR, Myc, PI3K‐Akt that viruses use or alter to reprogram nucleotide synthesis—to meet the increasing demand for these metabolites during infection. By organizing examples across virus families, we highlight the intersections between signaling cascades and metabolic pathways. In addition, we briefly discuss new insights into how these metabolic changes can impact host immune responses, highlighting the emerging field of immunometabolism.

While nucleotides, particularly pyrimidines, also contribute to a wide range of cellular processes—including epigenetic regulation, glycosylation, and ADP‐ribosylation—the current review is focused on their biosynthesis and the upstream signaling pathways that regulate nucleotide metabolism during viral infection. We acknowledge these other roles as important and emerging areas of interest, but they fall outside the scope of this review.

## 2 DNA Viruses

### 2.1 Herpesviruses

Herpesviruses are large dsDNA viruses encompassing eight known members that infect humans and many animals [45, 46]. Many members of the herpesvirus family increase the synthesis of pyrimidines and purines, which are critical for efficient viral replication. Cytomegalovirus, for instance, enhances pyrimidine flux, subsequently increasing levels of UTP, CTP, CDP, and UDP. This elevation in UDP levels is essential for the production of UDP sugars necessary for the glycosylation of virion proteins, thereby facilitating efficient replication. In addition, treatment with tunicamycin, an inhibitor of glycosylation, resulted in a 100‐fold reduction in infectious virions. This finding underscores the significant role of UDP sugars and consequently, glycosylation in the modification of viral proteins and their impact on the infection process [13].

In an interesting study, researchers found that UL97, a viral factor, can phosphorylate retinoblastoma (Rb) [47]. The phosphorylation of Rb enhances the production of nucleotide biosynthetic enzymes, which in turn leads to an increase in the synthesis of deoxynucleotide triphosphates (dNTPs). However, the study also revealed that there are other mechanisms through which HCMV can acquire dNTPs that do not depend on Rb. While UL97 plays a significant role in helping HCMV obtain nucleotides, it is not the only factor involved. It will be interesting to identify other factors that contribute to this process. In this study, the authors also showed that both salvage and de novo pathway enzymes play roles in HCMV replication, highlighting the lesser‐known role of the salvage pathway contributing to nucleotide synthesis.

Epstein–Barr virus (EBV) identified as the first tumor virus, effectively infects B cells and is known to be the causative agent of several malignancies like Burkitt lymphoma, Hodgkin lymphoma, and diffuse large B‐cell lymphoma [14]. EBV actively induces metabolic reprogramming of B cells. EBV induces the transition of B cells from a quiescent state to a state of hyperproliferation and requires nucleotide synthesis to facilitate this change. To meet the nucleotide demand, EBV stimulates mitochondrial one‐carbon metabolism, thereby providing essential one‐carbon units for nucleotide synthesis. The presence of Epstein‐Barr Nuclear Antigen 2 (EBNA2) is critical for the upregulation of the one‐carbon (1C) metabolic pathway. EBNA2 activates this pathway through a mechanism that involves Myc. Specifically, EBNA2 and Myc bind to the promoter region of the MTHFD2 gene, which encodes an enzyme integral to the 1C metabolism pathway, thereby enhancing the metabolic flux through this pathway. In this metabolic pathway, the cytosolic and mitochondrial enzymes MTHFD1 and MTHFD2 utilize the 5,10‐CH_2_‐THF substrate, derived from serine, to synthesize 5,10‐methenyl‐tetrahydrofolate and NADPH. This 5,10‐CH_2_‐THF is subsequently converted into 10‐formyl‐THF, which is necessary for purine synthesis [14].

Purine metabolism is also significantly activated during EBV infection in B cells, which is crucial in B cell immortalization. Furthermore, it has been established that the viral protein EBNA1 binds to both the ADA and AK4 genes. The alteration of the ADA gene within purine metabolism is critical, as B cells rely on this gene for their proliferation [15]. Pyrimidine metabolism also plays a crucial role in EBV infection, where CD19+B cells infected with EBV showed increased levels of pyrimidine enzymes CAD, DHODH, and UMPS proteins over a period of 28 days. In addition, the importance of pyrimidine synthesis during EBV infection is demonstrated by the observation that targeting pyrimidine metabolism through the inhibition of the enzyme DHODH significantly restricts the proliferation of EBV‐transformed B cells [48].

Furthermore, EBV has been demonstrated to upregulate both CTPS1 and CTPS2 enzymes, with CTPS2 exhibiting a partially redundant role with CTPS1 in EBV‐transformed cells. CTPS1 is essential for the proliferation of EBV‐transformed cells. These cells can compensate for deficiencies by relying on CTPS2 and salvage metabolism to synthesize CTP, which is critical for the synthesis of DNA, RNA, phosphatidylcholine, and phosphatidylserine. Notably, the addition of cytidine has been shown to rescue phenotypes that are deficient in CTPS1 and CTPS2. In addition, the expression of CTPS1 is regulated by key factors including EBNA2, Myc, and NF‐κB [16].

Furthermore, mutations in the de novo pyrimidine pathway enzyme CPTS1 are associated with severe EBV infections and the development of EBV‐related lymphomas, highlighting the role of nucleotide synthesis in viral infection outcomes and disease progression [16].

Kaposi's sarcoma virus (KSHV) is a pathogen that targets B cells and endothelial cells, resulting in a spectrum of malignancies [49, 50]. Research indicates that KSHV modifies central carbon metabolism, involving pathways such as aerobic glycolysis, glutaminolysis, and fatty acid synthesis. The virus reprograms metabolic processes either by directly regulating metabolic enzymes—such as the increased expression of glutaminolysis via glutamate receptors—or by modulating regulatory factors influencing metabolism, including Myc, AMPK, and HIF and mTOR [51].

The molecular intricacies of metabolic reprogramming remain only partially understood. Recent findings indicate that the viral protein vClycin interacts with cyclic‐dependent kinase CDK6, facilitating the phosphorylation of CAD at ser1900, which in turn activates CAD‐mediated pyrimidine synthesis. In addition, CAD has been shown to deaminate NF‐κb, thereby promoting aerobic glycolysis. The significance of pyrimidine synthesis is underscored by the evidence that the inhibition of both CAD and CDK6 leads to a reduction in KSHV replication and tumorigenesis [17]. In a separate study, the significance of asparagine and glutamine in providing the nitrogen needed for purine and pyrimidine synthesis during KSHV infection was highlighted. In addition, the study emphasized the important role of the purine and pyrimidine pathways in this context. It was found that blocking key enzymes, such as CAD of the pyrimidine pathway and phosphoribosyl pyrophosphate amidotransferase of the purine pathway, led to decreased cell proliferation [52]. These findings highlight essential molecular mechanisms that may inform potential therapeutic strategies against KSHV.

Upon the infection of quiescent human foreskin fibroblast (HFF) cells, cells infected with the herpes simplex virus type 1 (HSV‐1) exhibited an upregulation of intermediates in pyrimidine metabolism, specifically carbamoyl aspartate and deoxyuridine monophosphate (dUMP). The observed increase in UMP is likely attributable to the activity of two viral proteins, UL50 and UL2. The UL50 protein facilitates the production of heightened levels of dUMP while reducing the levels of deoxyuridine triphosphate (dUTP), thereby diminishing the incorporation of uracil into viral DNA. In contrast, UL2 is involved in the base excision repair mechanism of the HSV genome, facilitating the removal of uracil from the viral DNA.

Furthermore, it is plausible that the elevated levels of carbamoyl aspartate are influenced by signaling from growth factors [18]. The dependence on specific metabolic pathways varies based on the replication modes of the viruses. For example, HSV replicates at a faster rate than HCMV, resulting in minimal effects on glycolysis and fatty acid synthesis in HSV compared to HCMV. HSV predominantly utilizes the PPP and the tricarboxylic acid (TCA) cycle to produce the nucleotides necessary for its genome synthesis [53]. Interestingly, research indicates that during HSV‐1 infection, both arginosuccinate synthetase 1 (AS1) protein and mRNA levels decrease. AS1 is involved in the de novo synthesis of arginine, using aspartate in the process. Notably, knocking down AS1 mimicked the metabolic changes induced by wild‐type infection, which included an increase in nucleotides and their precursors. This highlights HSV‐1's reliance on altered host metabolism and underscores the significant role that a single metabolic enzyme plays in the virus's metabolic reprogramming [54].

Several interesting research opportunities persist in bridging the existing knowledge gap regarding herpesviruses and nucleotide reprogramming. A noteworthy finding is the identification of the viral factor vClycin encoded by KSHV, which plays a pivotal role in the activation of CAD, thereby underscoring the potential for targeting this viral protein in the development of novel antiviral therapeutics. A significant challenge in therapeutic targeting lies in the decision to focus on host versus viral proteins. While targeting viral enzymes may lead to a more rapid emergence of drug resistance, focusing on host metabolic enzymes—like those altered during CMV infection—may establish a higher evolutionary barrier for viruses to surmount in developing resistance [13]. Furthermore, considerable limitations persist in identifying the viral factors among the herpesviruses that contribute to the regulation of nucleotide metabolism, warranting further investigations.

Another promising avenue of research involves examining the role of salvage pathway enzymes, a pathway that has received less attention. Understanding whether this family of viruses employs distinct viral factors to regulate different branches of nucleotide metabolism—such as salvage versus de novo pathways—could yield critical insights. Such mechanistic elucidations may facilitate the advancement of more targeted therapeutic strategies in the treatment of herpesvirus infections (Table 1).

**Table 1:** Table highlights DNA and RNA viruses that regulate nucleotide metabolism highlighting the viral and host protein responsible for nucleotide regulation.

Viruses	Viral proteins	Host protein directly involved	Metabolites modulated upon infection	References
CMV	?	?	Increases levels of UTP, CTP, CDP, UDP	[13]
EBV	EBNA2	?	EBNA2 along with the Myc protein is involved in 1C metabolism necessary for purine synthesis	[14]
	EBNA1	ADA	EBNA1 binds to both ADA (directly involved) and AK4 (indirectly involved)genes in purine metabolism	[15]
	?	CTPS1 and CTPS2	Upregulate CTPS1 and CTPS2 of pyrimidine metabolism	[16]
1KSHV	vCyclin	CAD	CAD protein to regulate pyrimidine metabolism	[17]
HSV‐1	UL50 and UL2	?	Upregulation of pyrimidine metabolites‐ dUMP, dUTP, Carbomyl aspartate	[18]
Adenovirus	E4ORF1 and E4ORF6	?	Forms a complex with Myc and enables elevation of nucleotide levels	[19]
HPV	E6 E7	? ? RRM2	Indirect influence on nucleotide metabolism through glycolysis and Myc E6 influence nucleotide synthesis through growth factor signaling E7 is involved in the increase in availability of glutamine and enhances expression of genes in nucleotide metabolism E7 also enhances RRM2 levels, an enzyme that converts ribonucleotides to 2‐deoxyribonucleotides	[20, 21, 22]
VACV	VGF	CAD	Increase in pyrimidine metabolites	[23]
Polyomavirus	SV40T antigen	?	Nucleotide metabolism	[24]
HBV	?	?	Possible indirect alteration of nucleotide metabolism via Hbx	[25]
Influenza virus	? ?	? ?	Altered levels of ATP, ADP, AMP, GDP, CTP Inosine, hypoxanthine, AMP, urdine, cytidine Inosine, hypoxanthine, AMP, guanosine, hypoxanthine, uridine, cytidine	[26, 27, 28, 29, 30]
Murine norovirus‐1	?	?	IMP, hypoxanthine, xanthine, UTP	[31]
Newcastle disease virus	?		Indirectly influences nucleotide metabolites by enhancing one‐carbon metabolism	[32]
SARS‐Cov2	Nsp14 ORF9b and ORF3 Nsp10 Nsp15	IMPHD2 DCTPP1 TK1 TK1	Alters IMPHD2 to increase GTP levels DCTPP1, TK1, and CMAS are enzymes involved in nucleotide metabolism	[33, 34, 35]
HCV	?	?	Adenosine, inosine, GMP, UMP	[36]
Dengue virus	?	?	Inosine, adenine, guanine, guanosine, cytidine‐5‐monophosphate	[37]
HIV CAEV	? dUTPase	? UNG2	Influences nucleotide levels through effects on mTORC1 activity. Viral encoded dUTPase and mammalian cell encoded UNG2 maintains dUTP/dTTP ratios and prevents misincorporation of uracil in viral DNA.	[38, 39]

*Note:* ? symbol is used for viral proteins and host proteins that have not been identified. (The table highlights host enzymes that are directly involved in nucleotide metabolism.)

### 2.2 Adenoviruses

Adenoviruses which are characterized by their double‐stranded linear DNA genomes, which range in length from 25 to 48 kilobases and include inverted terminal repeats. These viruses infect a broad spectrum of vertebrates, encompassing mammals and fish, and are frequently utilized as vectors in gene therapy applications [55]. Enrichment of purine and pyrimidine pathways was observed, particularly at late time points of adenovirus infection in human lung fibroblast cells [56]. A notable gene product encoded by adenoviruses is E4ORF1, an oncoprotein that modulates host cell metabolism and signaling pathways. The E4ORF1 gene product comprises two domains: D2 and PBM. The D2 region exhibits homology with eukaryotic dUTPases, enabling interaction with E4orf6 to form a complex with Myc, thereby facilitating its activation. This activation enhances glycolytic processes and stimulates non‐oxidative phosphorylation, resulting in the synthesis of nucleotide intermediates.

Research indicates that adenoviruses harboring a mutant E4ORF1 demonstrate an inability to elevate nucleotide levels upon infection, in contrast to infections with wild‐type adenoviruses, thereby highlighting the role of E40RF1 in nucleotide synthesis [19, 57, 58]. A significant gap in knowledge is the need to further elucidate and identify adenovirus factors that contribute to metabolic reprogramming. It is important to determine whether these factors operate independently or synergistically to modulate nucleotide synthesis, as well as how these changes differ across various cell types. In addition, while studies indicate how various pathways contribute to the regulation of nucleotide levels, the mechanistic details of how purine and pyrimidine pathways are regulated during adenovirus infection remain to be elucidated.

### 2.3 Papillomaviruses

Papillomaviruses are a family of viruses characterized by small, circular double‐stranded DNA (dsDNA) genomes that infect a variety of hosts, ranging from mammals and reptiles [59]. Among these, the human papillomavirus (HPV) is particularly notable for its role in causing cervical cancer, which is predominantly transmitted through skin contact [60]. HPV‐related genital infections have also become the most prevalent sexually transmitted infections [59]. A comparative analysis involving four cervical cell lines and three cancer cell lines—comprising one HPV‐negative, two HPV‐positive, and one normal cell line—showed that HPV‐infected cancer cells displayed an increase in purine metabolites including metabolites of the salvage pathway [61]. Although alterations in nucleotide metabolism were observed in the other cell lines, the metabolic pathways upregulated by HPV appeared to be distinctly different from those in the HPV‐negative and normal cell line cohorts. This finding suggests that HPV induces unique metabolic modifications.

HPV encodes seven early proteins, among which E6 and E7 are recognized as the principal oncoproteins. These viral proteins interact with a variety of cellular targets to regulate metabolic processes, cellular signaling pathways, immune responses, and the inhibition of apoptosis [20, 21]. Distinct variants of HPV are known to induce the degradation of the tumor suppressor protein p53 via the formation of the E6‐E6AP‐p53 complex, subsequently promoting glycolytic activity. The degradation of p53 results in the activation of glucose transporters and various enzymes associated with glycolysis. Moreover, the E6‐E6AP‐p53 complex can bind to c‐Myc, thereby enhancing its activity and modulating the expression of genes implicated in metabolic functions, particularly those involved in nucleotide synthesis [61]. The E6 protein also activates key signaling pathways, such as the PI3K‐Akt pathway, which may lead to the activation of mTORC, as well as the MAPK pathway.

In addition, E7 facilitates the degradation of the retinoblastoma protein (pRb). This degradation not only increases the availability of glutamine for energy acquisition but also enhances the expression of genes involved in nucleotide metabolism, including dihydrofolate reductase, thymidylate synthase, ribonucleotide reductase, and thymidine kinase [62]. In addition, the regulatory subunit of ribonucleotide reductase complex (RNR) ribonucleotide reductase regulatory subunit M2 (RRM2) enzyme plays a crucial role in converting ribonucleotides into deoxynucleotides, which helps maintain the dNTP pools necessary for efficient nucleotide production. A study demonstrated that E7 enhances RRM2 levels through the ChK1‐E2F1‐DDR pathway, ensuring effective replication [22]. Moreover, E6 and E7 have been implicated in the activation of angiogenic factors, such as vascular endothelial growth factor (VEGF), while the E5 protein facilitates the activation of the epidermal growth factor receptor (EGFR). These interactions may contribute to alterations in nucleotide synthesis [63, 64, 65, 66].

Numerous studies highlight the roles of the E6 and E7 oncoproteins in regulating key metabolic pathways, including glucose, fatty acid, and amino acid metabolism. These pathways contribute to cancer progression and may indirectly increase nucleotide pools. However, there is limited information regarding the direct role of E6 or E7 in modulating de novo or salvage nucleotide‐specific pathways. In addition, gaining a better understanding of how specific metabolic enzymes within these pathways are regulated by HPV infection would be valuable for identifying drug targets. Therefore, further studies are essential to clarify the relationships among altered signaling pathways, disrupted energy metabolism, and their effects on nucleotide metabolism during HPV infection.

### 2.4 Poxviruses

The poxviruses comprise oval‐shaped viruses characterized by a double‐stranded linear DNA genome [67, 68]. Notable members of this family include the variola virus, which is the etiological agent of smallpox, and the monkeypox(mpox) virus, which is currently responsible for the ongoing mpox outbreak [69, 70]. Nucleotide metabolism is critically important for the efficient replication of the vaccinia virus (VACV), the prototype poxvirus. The inhibition of key enzymes in the de novo pyrimidine synthesis pathway—specifically, CAD, DHODH, and UMPS—has been demonstrated to reduce VACV replication [23, 71]. Moreover, treatment of infected primary fibroblast cells with nucleotide analogs, Trifluridine and Adefovir dipivoxil (ADP), resulted in a significant reduction in different poxviruses (VACV, mpox, and cowpox virus) replication, further emphasizing the importance of nucleotides during poxvirus replication and highlighting the potential of targeting nucleotide metabolism as a promising antiviral strategy [72]. In addition, VACV expresses the vaccinia growth factor (VGF), a cellular homolog of epidermal growth factor (EGF). VGF facilitates the proliferative effects of VACV by activating the EGFR pathway.

VGF is recognized for its role in the posttranslational regulation of CAD by phosphorylating the enzyme at serine 1859, a site that serves as a positive regulatory element for CAD. In experimental conditions where VGF was knocked out, the virus demonstrates a diminished capacity to upregulate metabolites within the pyrimidine synthesis pathways [73]. This observation highlights the critical role of VGF in the regulation of nucleotide synthesis during viral infection. The modulation of CAD by VGF is mediated through the mTORC1 signaling pathway. It was demonstrated that upon nutrient deprivation, VGF is required to phosphorylate CAD at serine 1859 to regulate pyrimidine synthesis. Moreover, VGF plays an important role in upregulating TCA cycle intermediates, that ultimately can supply metabolites necessary for nucleotide synthesis [74, 75].

In addition, VACV is known to alter other significant signaling pathways, including PI3K and Akt [41, 76]. Moreover, a late‐stage vaccinia protein, F17, has been identified as a disruptor of mTORC1 signaling; however, its specific role in the regulation of nucleotide metabolism remains to be determined [77]. Vaccinia virus, being a large virus, encodes over 200 proteins, including F17 and VGF. In addition to these proteins, poxviruses also encode viral factors that are directly involved in DNA replication. These proteins include thymidine kinase, thymidylate kinase, ribonucleotide reductase, and dUTPase. It remains to be determined whether these viral factors act independently or in synergy with other factors, such as VGF, in regulating nucleotide metabolism. Investigating the significance of these individual factors, along with VGF and F17, would provide a better understanding of their roles in reprogramming nucleotide metabolism. In addition, other members of this family, encode factors homologous to EGF; however, their specific roles in altering nucleotide metabolism within their respective viral contexts remain completely understudied and warrant further investigation [78].

### 2.5 Polyomaviruses

The polyomaviruses constitute a family of small DNA viruses that infect various vertebrate species [79, 80]. The capacity of polyomaviruses to induce tumors in murine models has been the subject of extensive investigation. The role of nucleotide synthesis during viral infection is highlighted by the inhibition of polyomavirus DNA replication using nucleotide analogs [81]. However, research on dissecting the mechanisms underlying nucleotide signaling and metabolism during infection has been limited. Research indicates that different members of the polyomavirus family employ the PP2A protein to activate distinct cellular signaling pathways. For instance, the small T antigen of polyomavirus (PyST) modulates PP2A activity to activate the MAPK signaling pathway, whereas the SV40 small T antigen influences the PP2A activities involved in the PI3K‐AKT pathway.

Furthermore, the SV40 T antigen is known to regulate a broad spectrum of biological processes, including cell cycle, apoptosis, and differentiation. Studies conducted with alphaT3 transgenic mice have revealed that the SV40 T antigen is integral to metabolic targeting, with substantial differences noted in nucleotide metabolism [24, 82]. SV40 T antigen is known to inhibit the function of p53, resulting in malignant cell proliferation. p53 plays a crucial role in the regulation of nucleotide metabolism and exerts a negative regulation on mTORC1 [83]. Evidence also suggests that SV40 T antigen activates the PI3K‐Akt signaling pathway. Given that mTORC1 is positioned downstream of AKT and considering the central role of mTORC1 in nucleotide metabolism, it would be worthwhile to explore the relationship between p53 dysregulation by SV40 T antigen and its effects on mTORC1 signaling, as well as the subsequent impact on nucleotide metabolism [84].

### 2.6 Hepadnaviruses

Hepatitis B virus (HBV)‐encoded protein HBx causes DNA damage and also alters nucleotide metabolism, and eventually leads to the development of Hepatocellular carcinoma [85, 86]. HBV is known to activate mTORC1 through HBx by inhibition of TSC1/2. HBx also modulates mTORC1 indirectly by activating p53, AFP, or Wnt/beta GSK3. Other proteins HBsAG also indirectly activate mTOR through the Wnt/beta GSK3 pathway [87]. HBV has been shown to induce nucleotide synthesis; however, the specific viral factor responsible for these alterations in nucleotides remains unknown. Given the important role of the viral protein HBx in altering host metabolic pathways, it is a potential candidate required to regulate nucleotide metabolism during HBV infection [25].

## 3 RNA Viruses

### 3.1 Orthomyxoviruses

This family of viruses comprises negative‐sense RNA, including several viruses capable of infecting humans [88]. The most prominent members of this family are the influenza strains that seasonally infect avians and humans. Influenza viruses are classified into three distinct types—A, B, C, and D—based on their nucleoprotein antigen. Primarily, influenza viruses exert their effects on the respiratory system, with pneumonia representing the most severe complication linked to this viral infection [88, 89, 90, 91, 92]. A comparative analysis of metabolites in cells infected by the human influenza strain A/PR/8/34 to those in uninfected cells revealed a notable decrease in ATP concentrations during the late phase of infection. This reduction was accompanied by a simultaneous increase in the levels of ADP and AMP. Furthermore, the concentrations of other nucleotides, specifically GMP and GDP, exhibited an increase similar to that observed for ADP and AMP, while CTP levels were found to be lower in the infected cells compared to the mock‐infected cells. In addition, the concentration of UDP‐GlcNAc, another product of nucleotide metabolism, increased significantly around 18 h postinfection in the infected cells. A similar trend was observed with another strain (RKI) [26].

In a recent study, the activation of the UMP synthesis pathway was observed in the infection of immune cells with Influenza virus, mostly likely to fuel the metabolite demand for the increase in RNA replication required by the virus [27]. Upon the infection of A549 cells with the H1N1 virus, it was observed that the virus modulates the metabolism of purines and pyrimidines. Specifically, metabolites associated with nucleotide metabolism, including inosine, hypoxanthine, and AMP, demonstrated significant upregulation at the 2‐h postinfection mark. In contrast, purine metabolites—namely inosine, guanosine, and hypoxanthine—and pyrimidine metabolites such as uridine and cytidine were downregulated at 5‐ and 8‐h postinfection. Overall, the metabolism of both purines and pyrimidines was markedly altered [28].

In another study, infection of mice with the H1N1/PR8 strain of influenza, led to enhanced purine degradation, with an increase in hypoxanthine, xanthine, and inosine indicative of GTP and ATP catabolism [29]. Other studies also highlight the elevation of purines and pyrimidines, for example, influenza infection affected lung metabolites, especially metabolites of purine and pyrimidine pathways [30]. In addition, in a separate study, significant alterations of purines and pyrimidines were identified in lung tissue upon infection with influenza in a mouse model at intervals of 0, 6, 10, 14, and 21 days. Influenza virus infection has been demonstrated to elevate the expression levels of mTORC1, Akt, and c‐Myc, all of which are established regulators of pyrimidine metabolism [93, 94, 95]. Additional research is necessary to elucidate the molecular mechanisms by which this virus, as well as other members of the orthomyxovirus family, can modulate nucleotide metabolism [96].

Recent research has predominantly focused on elucidating the alterations in nucleotide pathway‐related metabolite levels following influenza virus infection, marking preliminary efforts toward understanding the reprogramming of metabolism upon influenza infection. Nevertheless, several critical questions remain to be addressed. Specifically, the intricate molecular mechanisms by which influenza influences these targeted metabolic nodes, as well as the viral proteins implicated in the modulation of nucleotide levels, are not yet fully elucidated. A notable study has indicated that the hemagglutinin protein of the virus activates the mechanistic target of rapamycin complex 1 (mTORC1), whereas the M2 viral protein appears to downregulate the mTORC1 inhibitor REDD1 [94]. This raises the interesting prospect of conducting further investigations to determine whether these viral proteins also play a significant role in nucleotide metabolism.

An interesting example of how pyrimidines can have effects beyond genome synthesis was demonstrated when the pyrimidine inhibitor DHODH was used to block pyrimidine synthesis upon influenza infection. This inhibition reversed the suppression of mRNA nuclear export induced by the expression of NXF‐1, allowing for the expression of antiviral factors. This finding underscores the importance of a nuanced understanding of the unconventional effects that arise from modulating nucleotide metabolism. These effects extend beyond the established roles of pyrimidines in DNA and RNA synthesis and are crucial for uncovering the broader implications of targeting these metabolic pathways in therapeutic contexts [97].

### 3.2 Caliciviruses

Caliciviruses are small RNA viruses with non‐segmented, positive‐sense RNA, predominantly affecting various hosts from cattle to humans [98]. The human‐infecting members of this family, including Norovirus and Sapovirus, typically lead to acute gastroenteritis [98, 99, 100]. Upon infecting macrophages with murine norovirus virus (MNV)‐1, there was an increase in several metabolites from central carbon metabolism. These included upregulated metabolites of the nucleotide pathways such as IMP, hypoxanthine, xanthine, and UTP, and pathway products like UDP‐glucose and UDP‐D‐glucuronate [31]. In a follow‐up study on MNV infection, researchers extended their analysis to include two additional strains—CR3 and CR6—in addition to MNV‐1. They found that all three strains relied on glycolysis, glutaminolysis, and the PPP for efficient replication. Interestingly, only MNV‐1 exhibited a dependence on oxidative phosphorylation. These findings underscore the strain‐specific differences in metabolic reprogramming during infection and suggest that distinct metabolic dependencies could serve as potential targets for strain‐specific therapeutic intervention [101].

Furthermore, they identified viral protein NS1/2 as essential for glutaminase activity during the infection of macrophages by MNV. Glutaminolysis leads to the production of α‐ketoglutarate (α‐KG), which then enters the TCA cycle to generate metabolites necessary for nucleotide synthesis [102]. Given the identification of the viral proteins NS1 and NS2 as essential factors for glutaminolysis, it will be interesting to explore whether these viral proteins regulate nucleotide metabolism. In addition, there is evidence that MNV infection increases the levels of protein Akt; however, further links between this signaling pathway and alterations in nucleotide metabolism have yet to be established [103, 104].

### 3.3 Paramyxoviruses

This family of viruses comprises large, enveloped RNA viruses, many of which are pathogenic to humans, including mumps, measles, and the Nipah virus [105, 106]. Infection with Newcastle disease virus (NDV) results in the upregulation of intermediates involved in the synthesis of nucleotides, including ribose 5‐phosphate, glyceraldehyde 3‐phosphate, IMP, and UMP [32]. In addition, NDV infection enhances one‐carbon metabolism and increases the levels of intermediates associated with glycolysis. The findings further indicate that NDV relies on the oxidative branch of the PPP and upregulates the mitochondrial one‐carbon pathway to facilitate nucleotide synthesis. Moreover, the study identifies MTHD2, a known target of the MYC oncogene, as being upregulated to support nucleotide synthesis effectively [32]. However, the viral protein/s responsible for this regulation still need to be identified. Future investigations should prioritize the identification of viral factors and their interaction with metabolic pathways to advance the development of antiviral therapies.

### 3.4 Coronaviruses

Coronaviruses are enveloped positive‐sense RNA viruses [107]. Nsp14 is a viral protein associated with SARS‐CoV‐2, playing a critical role in the transcription and replication of viral mRNA. The expression of Nsp14 is known to increase the levels of genes involved in nucleotide metabolism. Research indicates that Nsp14 significantly alters the host transcriptome, with these changes mediated by IMPDH2, which catalyzes the biosynthesis of guanine nucleotides and results in increased GTP levels. Moreover, the application of IMPDH2 inhibitors appears to counteract the effects induced by Nsp14. Additionally, Nsp14 facilitates the activation of NF‐κB and CXCL8 expression, mediated through IMPDH2. Further investigations are warranted to ascertain whether additional cofactors are necessary to mediate the changes induced by Nsp14 within the context of SARS‐CoV‐2 [33]. Other viral proteins, including ORF9b and ORF3, are known to regulate DCTPP1, an enzyme facilitating the conversion of dCTP to dCMP. Furthermore, Nsp10 has demonstrated regulatory effects on TK1, while Nsp15 has been shown to regulate both CMAS and TK1. These proteins, TK1 and CMAS, are both integral to nucleotide metabolism [34].

Another study reported that differentially expressed genes were identified and pathway analysis was conducted on four different lung cell lines: NHBE, A549, A549. ACE2, and Calu3. The analyses revealed that the most significant pathways differed among the cell lines, with nucleotide metabolism showing the most pronounced alterations in the Calu3 cell line [35]. However, this further presents a challenge to identify factors contributing to cell context‐dependent regulation of specific metabolic pathways for efficient treatment. Future research would need to focus on elucidating the mechanistic foundations of these interactions. It is imperative to evaluate whether these metabolic pathways represent universal therapeutic targets or if they necessitate cell‐type‐specific intervention strategies.

### 3.5 Flaviviruses

Flaviviruses are small RNA viruses with genome sizes between 9 and 13 kb in length. The most famous members of this family that infect humans include the dengue virus, West Nile virus, yellow fever virus, and hepatitis C virus [108, 109]. In a comparison of Huh 7.5 cells with and without HCV infection, around 73 metabolites were found to be differentially regulated, with levels of adenosine, inosine, GMP, and UMP significantly increased. Also, metabolites of the PPP that feed into the nucleotide synthesis pathway, namely sedoheptulose‐7 phosphate and xylenate, were shown to be increased. Research in hepatitis C virus in hepatocytes showed alteration of major metabolic pathways with an increase in nucleotides in infected hepatocytes [36].

In a separate study involving HFFs infected with the Dengue virus, six metabolites related to nucleotide metabolism were found to be significantly upregulated at 10 h postinfection (hpi). These metabolites included inosine, adenine, guanine, guanosine, and cytidine‐5‐monophosphate, all of which showed increased levels compared to mock‐infected cells. However, at 12 and 48 hpi, there was a reduction in the number of upregulated nucleotide‐related metabolites. This highlights the importance of nucleotides during the earlier stages of virus replication, likely due to their necessity for genome replication [37]. An investigation into the varying levels of nucleotide metabolites during different stages of the viral life cycle could yield significant insights into their functional implications. It is essential to explore whether nucleotide metabolites play distinct roles at various phases of the viral life cycle. A comprehensive analysis is needed to enhance our understanding of these dynamics, which could ultimately inform the development of more effective antiviral strategies rooted in nucleotide metabolism [37].

### 3.6 Retroviruses

Retroviruses are characterized by the presence of reverse transcription, a process wherein the viral positive‐sense RNA genome serves as a template for the synthesis of dsDNA [110]. This DNA is subsequently integrated into the host genome, facilitating the viral replication cycle [110]. mTORC1 activity is crucial for the early replication of HIV. Inhibition of mTOR affects cellular metabolism and impacts the post‐entry steps of viral replication. The activity of mTORC1 plays a crucial role in regulating the synthesis of nucleotides that are essential for the reverse transcription process of HIV. Upon pretreatment with an mTOR inhibitor, there was a significant reduction in the levels of dNTPs, including dGTP, dCTP, TTP, and dATP. The study further investigated the role of specific metabolic enzymes within the pyrimidine pathway. The researchers reported that by blocking mTOR, the activation of CAD through its phosphorylation site at serine 1859 is inhibited, consequently decreasing the levels of pyrimidine metabolite intermediates, such as orotidine.

In addition, there were metabolites related to purine metabolism that were regulated via mTOR. The authors reported that inosine monophosphate dehydrogenase 1, the enzyme responsible for guanine‐containing purine synthesis, was dependent on mTOR activity. They also observed that RRMI (ribonucleotide reductase)—the enzyme that converts ribonucleotides to deoxyribonucleotides—was similarly dependent on mTOR [38]. This regulation occurs through an increase in glucose and c‐Myc uptake, both of which are influenced by mTORC1 activity. These processes contribute to elevated nucleotide pools that facilitate nucleotide synthesis [38]. Based on these studies, mTOR and its downstream pathways represent promising targets that can potentially be harnessed for therapeutic strategies. An intriguing approach would be to test combination therapies that target both mTOR and specific downstream nucleotide metabolic enzymes to evaluate their antiviral efficacy against HIV.

Another mode of regulating nucleotides is maintaining their balance, as an imbalance of nucleotides can be detrimental to virus replication. One such enzyme that plays an important role in regulating nucleotide levels necessary for viral replication is dUTPase. dUTP hydrolyzes dUTP into dUMP and pyrophosphate (PPi), with dUMP ultimately serving as a substrate for dTTP. By maintaining low dUTP/dTTP ratios, dUTPase prevents the misincorporation of uracil into DNA, thereby reducing the risk of mutations in the viral genome. In non‐primate lentiviruses, dUTPase is necessary for efficient viral replication in non‐dividing cells. However, viruses with defective dUTPase can still replicate in dividing cells, which have high endogenous dUTPase activity [39].

Regarding pathogenicity, the dUTPase of caprine arthritis encephalitis virus is necessary for the development of bilateral arthritis lesions in the carpus of infected goats. In contrast, dUTPase appears dispensable for pathogenesis of other non‐primate lentiviruses, such as visna virus and feline immunodeficiency virus). Primate lentiviruses do not encode their own dUTPase. Instead, they recruit the host cell enzyme uracil‐N‐glycosylase 2, which removes uracil from viral DNA. This mechanism prevents uracil misincorporation and is essential for maintaining viral replication fidelity [39].

## 4 Regulation of Immune Responses by Nucleotide Metabolism Upon Viral Infections

Our comprehension of nucleotide metabolism and the regulatory mechanisms associated with various viruses extends beyond the mere development of antiviral therapies. It is essential to recognize that targeting nucleotide metabolism may also present a double‐edged sword, as immune cells also require nucleotides and additionally use the same pathways to hijack nucleotide metabolism as virus‐infected cells [1, 111, 112, 113, 114, 115]. For example, the oncogene Myc is prominently recognized for its critical function in the regulation of nucleotide biosynthesis; however, it also plays an essential role in the metabolic reprogramming of T cells upon activation. Activated effector T cells engage in a variety of metabolic processes, including glycolysis, oxidative phosphorylation, and the PPP. Furthermore, they activate one‐carbon metabolism, with both PPP and one‐carbon metabolism serving as crucial mechanisms for nucleotide production. Notably, these pathways converge with those utilized by viruses during infection. Moreover, certain nucleotides possess immunoregulatory properties that can suppress immune responses, thereby creating an immunosuppressive milieu. This occurs through mechanisms such as the inhibition of immune cell differentiation mediated by adenosine. As a result, the targeting of virally infected cells necessitates careful consideration, as these interventions may profoundly impact the host immune response [114, 115].

There is a growing body of evidence regarding nucleotide imbalance and an increased expression of antiviral genes. The antiviral responses elicited by the inhibition of nucleotides are not exclusively a result of depriving the virus of its essential building blocks. Rather, the depletion of nucleotides also appears to induce an upregulation of the immune responses, thereby enhancing the overall antiviral effects. DD264, a compound targeting DHODH within the pyrimidine synthesis pathway, inhibits RNA virus replication by inducing the expression of interferon‐inducible genes. Consequently, it establishes a significant correlation between pyrimidine metabolism and the activation of the innate immune response [116, 117]. In analogous instances, SW835, a compound that inhibits the synthesis of pyrimidine, effectively blocks the replication of Ebola, vesicular stomatitis virus, and Zika virus [118]. In addition, it promotes the expression of interferon‐stimulated genes through the activation of the ATM kinase and the transcription factor IRF‐1.

Several other examples of depletion of pyrimidine pools induce host antiviral responses have been documented [119, 120]. In a distinct research investigation, an explanation was provided regarding the immune response prompted by pyrimidine deficiency. Findings indicated that diminished levels of pyrimidines induce the release of mitochondrial DNA, which subsequently activates the cGAS‐STING‐TBK1 signaling pathway. This phenomenon is attributed to the mitochondrial protease YME1L, which plays a crucial role in maintaining pyrimidine levels within the mitochondria by facilitating de novo nucleotide synthesis and the proteolytic degradation of the pyrimidine carrier SLC25A33. However, a decline in YME1L expression results in reduced pyrimidine levels, leading to the release of mitochondrial DNA into the cytosolic space and the subsequent activation of the cGAS pathway, thereby instigating an inflammatory response [117].

Consequently, it can be hypothesized that the upregulation of pyrimidine metabolism and the concomitant increase in pyrimidine levels during viral infections may serve to mitigate the immune response, thus assisting viruses in evading host defense mechanisms. A mechanism that focuses on the targeting of the enzyme CAD, which is essential for pyrimidine synthesis, has been found to restore the inflammatory response against SARS‐CoV‐2. Researchers have identified that the SARS‐CoV‐2 protein Nsp9 stimulates CAD, thereby facilitating pyrimidine synthesis, aerobic glycolysis, and purine synthesis in a manner dependent on CAD activity. In addition to CAD's role in regulating pyrimidine synthesis, it is also involved in the deamidation of RelA, which downregulates NF‐kB and attenuates the inflammatory response. Consequently, the inhibition of CAD restores the antiviral inflammatory response and decreases pyrimidine levels, making it an effective approach against SARS‐CoV‐2 [121].

It has been shown that the depletion of nucleotide pools during Hepatitis E Virus infection through inhibition of the pyrimidine and purine pathway was known to trigger ISGs independent of the canonical pathway JAK‐STAT1 that activates these ISGs. The Interferon Regulatory factors (IRFs) that are induced upon targeting nucleotide synthesis in the context of HEV infection include IRF1, DDX58, and IRF7, all of which are known to shave broad antiviral activity. However, the mechanism of how the depletion of nucleotides activates ISGs remains unknown [122].

## 5 Conclusion Remarks and Perspectives

The regulation of nucleotide synthesis is subject to stringent control, which significantly impacts the progression of the cell cycle [2]. Cells undergoing division must replenish nucleotides at a rate consistent with their division. The advancement of the cell cycle is intricately linked to the demand for nucleotides and the cell's capacity to acquire nutrients, thereby driving anabolic processes, including the synthesis of metabolites such as nucleotides. Moreover, conditions such as viral infections can exploit metabolic pathways that regulate nucleotide levels, thereby furthering viral propagation. The modulation of nucleotide levels occurs at the gene expression level, where various transcription factors are involved, and at the substrate level, which is essential for the modulation of the enzymes necessary for nucleotide synthesis.

While the comprehensive regulation of nucleotides falls beyond the scope of this review, it is important to note the involvement of several transcription factors in this process. For instance, Myc has been linked to uncontrolled cellular proliferation when either overexpressed or mutated. Myc is associated with the long‐term regulation of nucleotide synthesis, particularly in coordination with kRas [5, 123, 124, 125]. In addition, the Myc‐Eif4e complex is known to regulate the mRNA translation of phosphoribosyl‐pyrophosphate synthetase 2 (PRPS2) [126]. Other long‐term regulatory factors that integrate cell signaling to modulate nucleotide metabolism include mTORC1, p53, LKB1, PTEN, and YAP1 [127, 128, 129, 130, 131].

Short‐term regulation, which involves direct activation of metabolic enzymes, includes factors such as ERK, PI3K, mTORC1, and SIRT3 [5, 11, 132, 133, 134, 135, 136]. Whilst we know that several viruses upon infection can cause an increase in these long and short‐term factors and regulators of signaling like mTORC1, there are still significant gaps in our understanding of established links between signaling and altering of nucleotide levels. For example, late protein F17 of vaccinia virus has been shown to dysregulate mTORC1 signaling. However, whether the F17 protein affects nucleotide levels due to mTORC1 dysregulation would need to be elucidated [77].

Some viruses directly encode proteins that play a role in nucleotide metabolism. For example, HSV and VACV, both encode the enzyme thymidine kinase, which is directly involved in nucleotide regulation [137, 138]. However, there is a further need to identify viral proteins of several other viruses that have no annotated function related to nucleotide metabolism but can potentially directly influence nucleotide metabolic enzymes. Further investigation is imperative not only to determine whether a specific viral protein is crucial for the regulation of a metabolic pathway but also to clarify its functional role at the molecular level and to explore the underlying mechanistic insights of its actions.

An additional critical aspect to consider regarding nucleotide regulation involves the often‐neglected salvage mechanism of restoring nucleotide levels. Recent research focusing on cancer has demonstrated that both de novo synthesis and the salvage pathway play significant roles in supporting tumor growth, thereby highlighting the often‐overlooked importance of the salvage pathway in enhancing nucleotide availability [139]. Therefore, in conditions such as viral infections, which, like cancer cells, have a high nucleotide demand, it becomes essential to analyze the contributions of the salvage pathway as well as the feedback mechanisms that fuel the nucleotide synthesis pathways to gain a deeper understanding of how nucleotides are regulated during viral infections.

Importantly, metabolism is a dynamic process heavily influenced by its physiological context [140]. The specific tissue under study, stress and genetic factors influence metabolic outcomes. A significant limitation in the investigations of metabolic regulation by viruses pertains to the methodologies and tools utilized in the study of metabolism. Although an increasing number of research groups are dedicated to identifying and elucidating the pathways through which viruses modulate metabolic processes, it is crucial to underscore the necessity of conducting in vivo research. This is vital, as metabolic responses in vivo can differ markedly from those observed in vitro. To uncover novel antiviral targets effectively, a comprehensive understanding of in vivo metabolism is imperative for identifying specific metabolic nodes amenable to therapeutic intervention. It is essential to advocate for further studies utilizing stable isotope tracing and metabolic flux analysis within appropriate in vivo infection models.

In addition, the application of spatial metabolomics holds great promise for elucidating how various viruses rewire metabolic pathways within distinct localized regions of specific tissues [141, 142, 143]. Current studies indicate that certain viruses can regulate or uncouple the central metabolic regulator mTOR in response to nutrient scarcity, thereby facilitating replication under conditions of nutrient limitation. Incorporating spatial metabolomics into this study framework could yield deeper insights into how different areas within a tissue, characterized by nutrient‐rich versus nutrient‐depleted environments, differentially influence viral metabolic regulation.

For viral infections, the type of virus infecting would result in different and distinct modes of metabolic regulation. DNA viruses have been documented to interact simultaneously with various cellular and metabolic processes upon infection. In contrast, RNA viruses exhibit a more selective interaction with cellular proteins [144]. Therefore, each type of virus might have different modulatory pathways to regulate metabolism, the details of which need to be elucidated for therapeutic advancement. Furthermore, in addition to the intrinsic genomic characteristics of the virus, evidence indicates the existence of strain‐specific differences among various viral strains, each exhibiting unique metabolic preferences. It is also important to consider the specific stage of the viral life cycle when investigating particular metabolic vulnerabilities. Different phases of the cycle may require distinct metabolic pathways, as the viral needs for specific metabolites can vary at different stages essential for effective replication and propagation. When studying metabolic rewiring induced by viral infections, it is imperative to account for factors such as the virus types, their morphological and structural features, host specificities, various viral strains, and the stage of replication. These elements are vital for a comprehensive understanding of the metabolic dynamics at play during viral infections.

In addition, the link between immune regulation and nucleotide metabolism serves as an exciting avenue to pursue in terms of antiviral therapy. Though there are several reports of a link established between the generation of antiviral response upon disruption of pyrimidine levels, the molecular mechanisms remain known. Given the importance of immune cell response upon infection, an understanding of the molecular details of these findings would help advance antiviral therapy greatly.

The targeting of nucleotides has been one of the most common classes of drugs and there are multiple excellent reviews covering the mechanisms targeting these viruses through these drug classes that are beyond the scope of this review [1, 44, 141, 142, 145, 146, 147, 148]. However, we would like to emphasize that combining the targeting of nucleotides with the inhibition of the signaling pathway can be a better therapeutic strategy and that we must aim to understand the molecular underpinnings underlying the alteration of nucleotide metabolism to advance antiviral therapeutic strategies.

## Author Contributions

**Zhilong Yang:** conceptualization, formal analysis, funding acquisition, project administration, supervision, visualization, writing – review and editing. **Lara Dsouza:** conceptualization, formal analysis, visualization, writing – original draft, review, and editing.

## Conflicts of Interest

The authors declare no conflicts of interest.

## Data Availability

Data sharing not applicable to this article as no data sets were generated or analyzed during the current study.
